# Human papillomavirus (HPV) 16 infection is not detected in rectal carcinoma

**DOI:** 10.1186/s13027-020-00281-z

**Published:** 2020-03-05

**Authors:** Sandra F. Martins, Vânia Mariano, Mesquita Rodrigues, Adhemar Longatto-Filho

**Affiliations:** 1 Life and Health Science Research Institute (ICVS), School of Health Sciences, School of Medicine, University of Minho, Campus de Gualtar, 4710-057 Braga, Portugal; 2 ICVS/3B’s-PT Government Associate Laboratory, Braga/Guimarães, Braga, Portugal; 3 Coloproctology Unit, Braga Hospital, Braga, Portugal; 4 Molecular Oncology Research Center, Barretos, São Paulo, Brazil; 5 Laboratory of Medical Investigation (LIM) 14, Faculty of Medicine, University of Sao Paulo, Sao Paulo, Brazil

**Keywords:** Rectal cancer, Human papillomavirus 16, Carcinogenesis

## Abstract

**Introduction:**

Persistence of human papillomavirus (HPV) infections is associated with squamous cell carcinomas of different human anatomic sites. Several studies have suggested a potential role for HPV infection, particularly HPV16 genotype, in rectal cancer carcinogenesis.. The aim of this study was to assess the frequency of oncogenic HPV 16 viral DNA sequences in rectal carcinomas cases retrieved from the pathology archive of Braga Hospital, North Portuga.

**Methods:**

TaqMan-based type-specific real-time PCR for HPV 16 was performed using primers and probe targeting HPV16 E7 region.

**Results:**

Most of the rectal cancer patients (88.5%, *n* = 206 patients), were symptomatic at diagnosis. The majority of the lesions (55.3%, *n* = 129) presented malignancies of polypoid/vegetant phenotype. 26.8% (*n* = 63) had synchronic metastasis at diagnosis. 26.2% (*n* = 61) patients had clinical indication for neoadjuvant therapy. Most patients with rectal cancer were stage IV (19.7% patients), followed by stage IIA (19.3%) and stage I (18.5%). All cases of the present series tested negative for HPV16.

**Conclusion:**

The total of negative tests for HPV 16 infection is a robust argument to support the assumption that HPV 16 infection, despite of previous evidences, is not involved in rectal cancer carcinogenesis and progression.

## Introduction

Human papillomavirus (HPV) is one of the most common sexually transmitted infections and its relationship with some cancer types has been well established [1–3]. Persistent infections can progress to premalignant and malignant lesions [4] and approximately 4.8% (610,000 cases) of all cancers worldwide are attributed to HPV infection [5].

A strong causal relationship has been demonstrated between HPV and some types of cancers such as cervix uteri, penis, vulva, vagina, anus and oropharynx [4, 6]. Controversial results have been obtained regarding the role of HPVs in esophagus [6–8], oral cavity [6, 9], breast [10–12] and lung cancers [13, 14]. In cervical lesions the HPV DNA is frequently found integrated into the host genome particularly in high grade cervical intraepithelial neoplasia and invasive cancer cases [15, 16]. This type of cancer is recognized to develop through a multistep process, from premalignant lesions, cervical intraepithelial neoplasia (CIN, graded 1–3 according to severity) into cancer [17]. In oropharyngeal squamous cell carcinoma (OPSCC) besides being a well-known risk factor, HPV also is related with improved survival [18–22]. Moreover, HPV is detected in 80–90% of anal cancer cases, mostly HPV 16; The high frequency of HPV16 infection in this anatomic region possibly reflects a differential tropism of HPV 16 or an increased probability of HPV 16 to lead to malignant transformation in the anal mucosa [23, 24]. Not surprisingly, in recent years, a growing number of studies suggest a potential role of persistent HPV infection also in colon and rectal carcinogenesis [25], but the association between HPV and rectal cancer (RC) remains controversial and inconclusive since HPV detection rate ranges from 0 to 84% [25–27]. Despite of several studies have focused on the association of HPV and RC, few reports dedicate a large study on rectal cancer, particularly. Based on this lack of precise information about this topic, we sought to investigate the frequency of HPV 16, and its potential association with rectal carcinoma. The current study was designed to specifically assess the incidence of HPV16 viral DNA sequences in rectal cancer carcinomas derived from a series of RC patients treated at Braga Hospital, North Portugal.

## Methods

### Rectal tumor series

Data from 233 patients with confirmed rectal cancer treated in Hospital de Braga, Portugal, from 2005 to 2010 were collected prospectively. Tumor localization was recorded and classified as rectum, when tumors localized between anal verge and 15 cm at rigid rectoscopy [28].

The histological type of rectal cancer was classified by an experienced pathologist (FP) and tumor staging was graded according to the TNM classification, sixth edition [29].

From the initial group of 233 rectal cancer cases, patients with tumor localized at the proximal rectum (*n* = 49) and patients submitted to neoadjuvant therapy (*n* = 40) were excluded from the study. Thus, tissue samples from 144 patients were performed for DNA extraction and HPV analyses.

### Processing of tumor samples and DNA extraction

The DNA was extracted from formalin fixed paraffin embedded (FFPE) samples using the DNA QIAamp Micro Kit (Qiagen, USA) according to manufacturer’s instructions. Briefly, 4–6 formalin-fixed, paraffin-embedded 10-μm sections were submitted to deparaffinization of sections with 100% xylene, followed by washes of ethanol (100, 90, and 50%) and incubation with DNA extraction buffer overnight. All DNA samples were quantified using NanoDrop 2000 (ThermoScientific) and maintained at − 20 °C until use.

### HPV analysis

TaqMan-based type-specific real-time PCR for HPV 16 was performed using primers (forward 5′-GATGAAATAGATGGTCCAGC-3′ and reverse 5′- GCTTTGTACGCACAACCGAAGC-3′) and TaqMan probe (5′- 6FAMCAAGCAGAACCGGACAG-MGBNFQ-3′) E7 region of HPV type 16. The reaction mixture was prepared as follows: TaqMan Universal PCR Master Mix 1 x (Applied Biosystem, Inc., EUA), 400 nM of each primer, 200 nM TaqMan probe and water DNAse and RNAse free. PCR amplifications were carried out after the addition of 5 μl of the sample containing template DNA in a final volume of 25 μL of the PCR reaction mixture. The amplification conditions were as follows: initial denaturation for 10 min at 95 °C, followed by 40 amplification cycles of 15 s each at 95 °C and 1 min at 60 °C (annealing-extension step). Each PCR reaction included negative (water) and positive controls (DNA extracted from cell CasKi). All samples and controls were tested in duplicate and were considered positive when both replicated amplified in a cycle < 38 [30].

As a positive control for the quality of the template DNA, primers for β-globin gene were included in the reactions, as described previously [7].

## Results

### Rectal cancer patient’s characterization

The characteristics of the in Table 1 and previously published [31]. Of the 233 rectal cancers patients, most (50.6%, *n* = 118), were localized in the middle third, followed by distal rectum, 28.3% (*n* = 66), and proximal rectum in 21% (*n* = 49).

**Table 1:** The main characteristics of rectal cancer treated at Hospital de Braga between 2005 to 2010 are depicted

Characteristics	Rectum n (%)
Macroscopy	
Polypoid/vegetant	130 (55.8)
Ulcerated Infiltrative	49 (21.0)
Exofitic	25 (10.7)
Villous	21 (9.0)
No information	8 (3.4)
Histological Staging	
0	21 (10.3)
I	38 (18.7)
IIA	43 (21.2)
IIB	0 (0.0)
IIIA	12 (5.9)
IIIB	31 (15.3)
IIC	13 (6.4)
IV	37 (18.2)
Serosal involvement	
With	109 (53.7)
Without	70 (34.5)
No information	24 (11.8)

Most of the rectal cancer patients (88.5%, *n* = 206 patients), and the instrumental diagnosis performed by by total colonoscopy in 79.8% (*n* = 186) and rectosigmoidoscopy in 18.9% (*n* = 44). In 1.3% (*n* = 3) it was impossible to perform an endoscopic exam (rectal stenosis).

The majority of the lesions (55.3%, *n* = 129) presented malignancies of polypoid/vegetant phenotype. The remaining 21.0% (*n* = 49) were ulcerated lesions, and 10.7% (*n* = 25 showed an infiltrative pattern; 9.0% (*n* = 21) were exofitic cancers; 0.4% (*n* = 1) were villous aspect and for the reminder seven patients (3%) we did not have macroscopic appearance information.

Sixtythree out of 233 (26.8%) had ....had synchronous metastasis at diagnosis, more frequently lymph node (10.2) and hepatic metastasis (8.5%). Pelvic magnetic resonance (MR) and rectal endoscopic ultrasound (EUS) were used combined for local staging. After staging, 26.2% (*n* = 61) patients had indication for neoadjuvant therapy.

Finally, postoperative histological staging, graded according to the TNM classification, seventh edition (American Joint Committee on Cancer - AJCC). Most patients with rectal cancer were stage IV (19.7% patients), followed by stage IIA (19.3%) and stage I (18.5%).

### HPV detection

Patients with carcinoma localized at the proximal rectum (*n* = 49) and patients submitted to neoadjuvant therapy (*n* = 40) were excluded from the initial group of 233 rectal cancer cases. Thus, tissue samples from 144 patients were subjected to DNA extraction and HPV analysis. None of the 144 samples analyzed for HPV16 E7 DNA tested positive. All cases were unequivocally negative.

## Discussion

The growing evidences that HPV is associated with carcinogenic processes of squamous cell carcinomas arising at different anatomical sites stimulated several researches to investigate the presence of HPV infections in different tumours types. Indeed, viral infection are responsible for about 15% of cancers, mainly by favoring genetic instability and inducing chromosomal aberrations [31–33]. The role of HPV in cancer development has already well documented for cervix uteri, penis, vulva, vagina, oropharynx, and anal canal cancers worldwide. However, there are scarce data concerning the relationship between HPV and rectal cancer in Portugal [4, 6, 23, 24].

Despite colonrectal is the third most common type of cancer [34–37] and the fourth most frequent cause of cancer death [34–38], in Portugal, this neoplasia is the second most frequent cancer and the second leading cause of cancer death in both genders [39]. About rectal cancer, in particular, the district of Braga exhibited incidence of 16.8/100,000 inhabitants in 2008 [40], which is substantial prevalence taking in account that Braga population is currently 136,885 (official information from Braga Council of the urban perimeter, 2011) people.

Most of CRCs are demonstrated to developed from a benign pre-neoplastic lesion (polyp).. The transition from normal mucosa to malignancy, through the adenoma-carcinoma sequence, usually takes ten or more years and is characterized by the change in innumerable genes associated with the maintenance of cellular homeostasis [41, 42]. Internal factors such as personal or family history of CRC and/or polyps, personal history of inflammatory bowel disease and hereditary genetic conditions confer a higher risk of developing CRC [42, 43]. However, many cases can be associated to a plethora of environmental and compartmental factors such as sedentary lifestyle, obesity, long-term smoking, excessive alcohol consumption and diet [42, 44, 45]. Among external factors, HPV has emerged as an important variable be considered for many carcinomas. HPV infection association with colon and rectal cancer is particularly contentious [6] and doubtful etiological source of CRC, since HPV detection rates range from 0 to 84% [46–50]., Most of these studies, however, did not take into account the tumor localization, and/or included series of malign and benign forms of cancer). Also, several reports propone a positive association and suggest a possible role of HPV in colorectal cancers development [46, 48, 49, 51] but non-clarifying the nature of the association [52]. A possible relationship between HPV and RC, could represent an impact in the global incidence of this cancer, as it could be possible to implement strategies for screening and control this malignancy through HPV testing.

Variation of HPV infection frequencies in rectal cancer is extraordinarily high, as anticipated, probably due to differences in methodologies used, preservation of the samples in retrospective series and some particular characteristics of populations [23]. Despite the controversies, is far to be proved the direct relationship between HPV and rectal cancer, as occur with cervical cancer [53]. The data available are controversial and incontestably intriguing. Our study has an important limitation because we opted to use only sequences for HPV 16 test because it is the most common type in various anatomic sites worldwide [24]. The results we achieved exclude the possible role of this HPV 16 in rectal carcinogenesis, which is endorsed by others [53]. These data, however, encourage further studies, with larger series and involving other segments of colon aiming to investigate the presence of other types of HPV.

## Data Availability

Please contact author for data requests.

## Abbreviations

CIN: Cervical Intraepithelial Neoplasia
CRC: Colorectal Cancer
HPV: Human papillomavirus
IARC: International Agency for Research on Cancer
OPSCC: Oropharyngeal Squamous Cell Carcinoma
RC: Rectal Cancer
